# Planning and performing CT-guided interventions for source control in septic patients: a survey by the European Society of Emergency Radiology

**DOI:** 10.1186/s13244-026-02374-6

**Published:** 2026-09-15

**Authors:** Anne Frisch, Maria Isabel Opper Hernando, Kerstin Rubarth, Ann-Christine Stahl, Ana Blanco Barrio, Raffaella Basilico, Vittorio Miele, Francesca Iacobellis, Myrto Bolanaki, Denis Witham, Marc Dewey, Julian Pohlan

**Affiliations:** 1 Department of Diagnostic and Interventional Radiology, Klinikum Lippe, Bielefeld University, Medical School and University Medical Center OWL, Detmold, Germany; 2 Department of Radiology, Charité – Universitätsmedizin Berlin, Corporate Member of Freie Universität Berlin and Humboldt-Universität zu Berlin, Berlin, Germany; 3 Institute of Biometry and Clinical Epidemiology, Charité – Universitätsmedizin Berlin, Corporate Member of Freie Universität Berlin and Humboldt-Universität zu Berlin, Berlin, Germany; 4 Department of Radiology, University of Cambridge, Cambridge University Hospitals, Cambridge, United Kingdom; 5 Department of Radiology, Emergency Section, Hospital Universitario Morales Meseguer, Murcia, Spain; 6 Department of Medical, Oral and Biotechnological Sciences, University G. d’Annunzio Chieti-Pescara, Chieti, Italy; 7 University of Florence and Department of Radiology, Careggi University Hospital, Department of Biomedical Experimental and Clinical Sciences, Florence, Italy; 8 Department of General and Emergency Radiology, Antonio Cardarelli Hospital, Naples, Italy; 9 Department of Emergency and Acute Medicine, Charité – Universitätsmedizin Berlin, Corporate Member of Freie Universität Berlin and Humboldt-Universität zu Berlin, Berlin, Germany; 10 Department of Cardiology, General Internal Medicine and Conservative Intensive Care Medicine, Vivantes Klinikum Am Urban, Berlin, Germany; 11 Department of Radiology, Academy of Medical Sciences, University of Cambridge, Cambridge, United Kingdom; 12 Berlin Institute of Health, Charité – Universitätsmedizin Berlin, Corporate Member of Freie Universität Berlin and Humboldt-Universität zu Berlin, Berlin, Germany; 13 UCB Pharma GmbH, Monheim am Rhein, Germany; 14 Fliedner Fachhochschule Düsseldorf, University of Applied Sciences, Düsseldorf, Germany

**Keywords:** CT-guided interventions, Sepsis, Emergency radiology, Source control

## Abstract

**Objectives:**

To analyze emergency radiologists’ perspectives on computed tomography (CT)-guided interventions for source control in patients with sepsis.

**Materials and methods:**

In 2023, members of the European Society of Emergency Radiology (ESER) were surveyed on the use of CT in the management of sepsis (*n* = 297). The questionnaire included demographic details, clinical experience, and questions on the timing and justification for CT. In a secondary analysis focused on CT-guided interventions, the obtained data were compared to results from a previous single-center survey among clinicians and general radiologists (*n* = 719). Responses were gathered anonymously, and data were analyzed using descriptive statistics and chi-square tests.

**Results:**

The perspectives of 144 emergency radiologists were compared with those of 719 participants from various specialties. Most respondents, i.e., 85.3% (*n* = 58) of emergency radiologists, strongly agreed on the importance of CT for planning interventions in patients with sepsis. Preferences for the timing of CT-guided intervention leaned towards 1–6 h among emergency radiologists (66.7%, *n* = 36) and clinicians (64.1%, *n* = 211), while a majority of general radiologists accepted 6–12-h (58.3%, *n* = 14). Professional experience did not affect the agreement on the importance of CT among emergency radiologists (board-certified, 85.7% (*n* = 30) vs senior, 84.8% (*n* = 28)) or timing preferences (board-certified, 60.7% (*n* = 17) vs senior, 73.1% (*n* = 19)).

**Conclusion:**

The ESER survey confirmed the importance physicians attach to CT for planning interventions in patients with sepsis. While preferences for intervention timing varied slightly among specialties, there was a wide agreement on the importance of prompt CT-guided interventions within the first 12 h of focus detection.

**Key Points:**

***Question*** What is the expert assessment on the significance of CT imaging for procedure planning and on the ideal timing of CT-guided interventions in sepsis.

***Findings*** the surveyed experts value CT for planning interventions in patients with sepsis, and a prompt CT-guided intervention ideally within the 6 h of focus detection.

***Critical relevance statement*** specialized imaging techniques and minimally invasive treatments for source control become more widely used. The perspective of emergency radiologists compared to that of general radiologists and clinicians helps to weigh the role of CT-guided interventions in septic patients.

**Graphical Abstract:**

**Figure:**
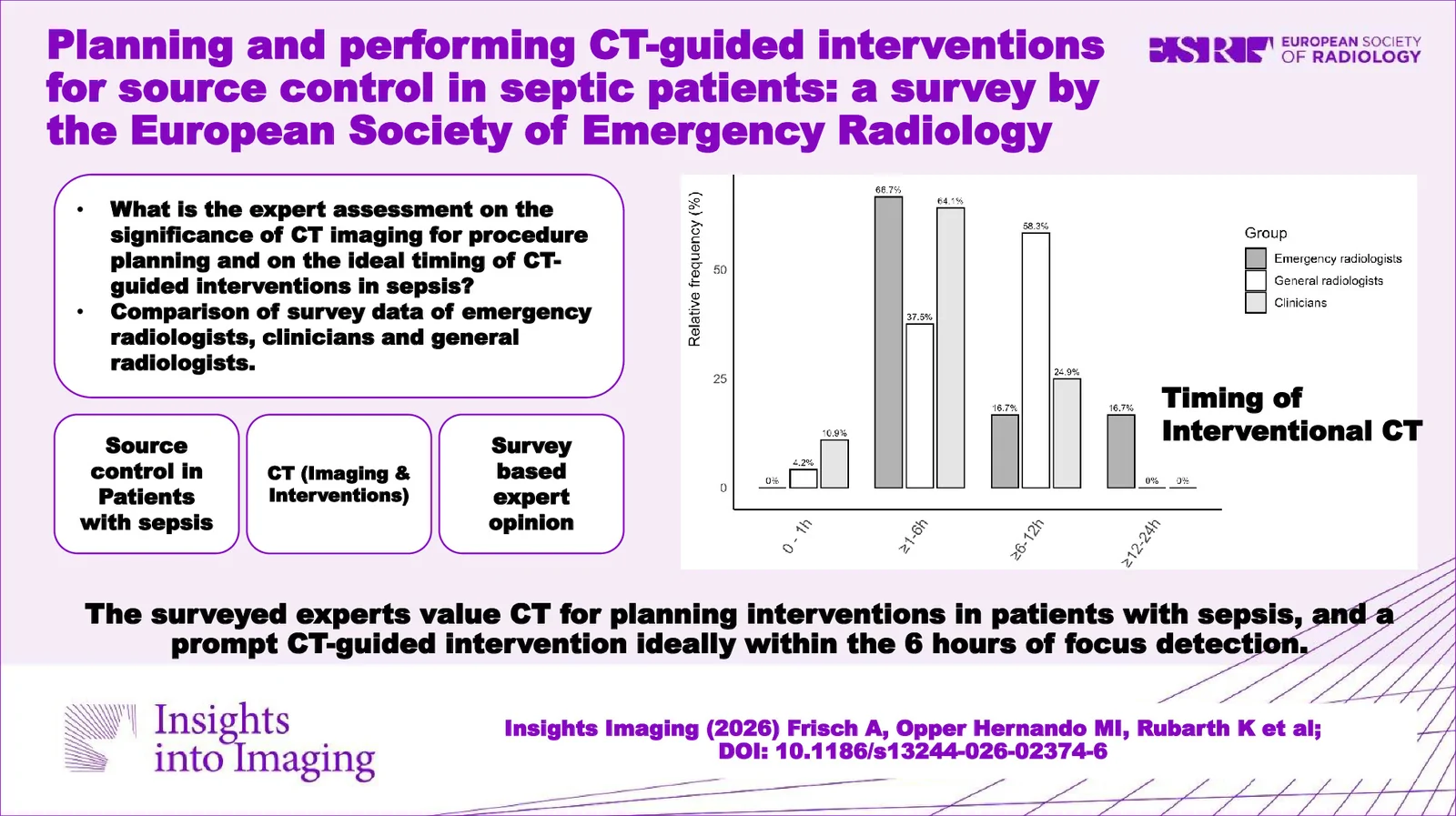

## Introduction

Sepsis is characterized by organ failure due to the body’s response to infection and is associated with considerable mortality [1, 2]. Early identification and treatment are essential for outcome improvement [3], as emphasized by global consensus guidelines [1, 3–7].

While sepsis guidelines recommend rapid identification of anatomical sources of infection for source control, they provide no specific guidance on the use of computed tomography (CT) [1, 4, 5]. Importantly, several studies indicate that CT plays a vital role in verifying suspected infection sites and can thus contribute to decision-making regarding medical, surgical, and interventional management [8–14].

CT-guided interventions have become a relevant therapeutic option in the management of localized infections [15, 16]. These offer minimally invasive source control even in critically ill patients, when surgery is not the first choice. Yet, when it comes to patients with sepsis, there is no consensus regarding indication, timing, and procedural strategies [5]. The above-quoted guidelines make no recommendations on CT-guided interventions in patients with sepsis.

We previously surveyed perspectives of final-year medical students and physicians from various specialties on the use of CT in patients with sepsis [17–19]. Even though abscess drainage is generally safe and efficacious [15, 16], there is a lack of evidence on CT-guided interventions for managing infectious foci in patients with sepsis.

Specialized imaging techniques for critically ill patients are becoming more widely used in many institutions. Therefore, it is important to gain insights into emergency radiologists’ attitudes and practices on imaging-derived management strategies for sepsis. This study aims to explore the role of CT-guided interventions in septic patients from the perspective of emergency radiologists, with a secondary analysis and comparison of general radiologists and clinicians.

## Materials and methods

### Survey

All members of the European Society of Emergency Radiologists (ESER) were invited to participate in our survey. We selected this cohort to represent the perspectives of radiologists with expertise in the field. Membership in the ESER served as an indicator that respondents were actively engaged in emergency radiology practice. Results are reported in line with the “Strengthening the Reporting of Observational Studies in Epidemiology (STROBE)” guidelines pertinent to cohort studies. The local ethics review board approved the study (reference number EA1/203/21), which was carried out in accordance with the Declaration of Helsinki. Data from a previously published analysis [17, 19] were included for comparing the attitudes and practices of emergency radiologists with those of other clinicians and general radiologists. The methodology and survey results pertaining to the diagnostic use of CT in septic patients have been published before [17, 19]. Results from questions that cover the role of CT-guided interventions in the treatment of septic patients are presented here. According to ESER, *n* = 297 were active members of the ESER at the time of the survey, of those *n* = 144 participated in the survey, i.e., a 48% participation rate. Participation was noted from > 20 European countries, with Italy, the United Kingdom, Turkey, Germany, and Spain being most common in descending order.

### Questionnaire structure

An interdisciplinary team created and approved a questionnaire focusing on the use of CT scans in patients diagnosed with sepsis. After validating its applicability in a cohort of final-year medical students and in physicians from a prominent tertiary care facility, the questionnaire was adapted to be used in the ESER cohort. While its basic structure is similar, some minor adjustments were made to demographic questions and CT timing [17, 19]. The questionnaire was subdivided into several sections. Initially, participants were asked to consent to the data protection declaration and whether they previously participated in the survey. Demographic information, including hospital rank (resident, board-certified, senior, or chief physician), years of post-licensure experience, medical specialty, any relevant fellowships related to sepsis, and the main location of employment, was gathered. Resident physicians were defined as licensed doctors undergoing residency training for board certification. The group of clinicians included surgeons, internal medicine physicians, and anesthesiologists, which were analyzed in more detail elsewhere [17]. Participants were also asked whether they were directly managing septic patients. To assess their views on CT for septic patients, responses were captured on a 4-point Likert scale: (1) strongly disagree, (2) somewhat disagree, (3) somewhat agree, and (4) strongly agree. Additionally, they stated their professional opinion on the preferred timing of CT interventions after focus identification from four options: (1) less than 1 h, (2) 1–6 h, (3) 6–12 h, and (4) 12–24 h. The access link to the online survey was sent to all ESER members by email. Each participant was permitted to respond only once.

### Data analysis

Data were gathered anonymously using LimeSurvey (version 5.3.25, 2022; LimeSurvey GmbH). The variables analyzed included baseline characteristics such as (a) years of experience, (b) workplace, and (c) medical specialty, as well as factors about the benefits and risks associated with CT scans in septic patients. Since not all items were mandatory, sample sizes may vary across items. Accordingly, all results are reported along with sample sizes. Descriptive statistics and chi-square tests were performed using R (version 4.4.1), with the significance level set at α < 0.05. Data analysis was exploratory. Therefore, no adjustment for multiple testing was performed. The results should be viewed as hypothesis-generating.

## Results

**Fig. 1:**
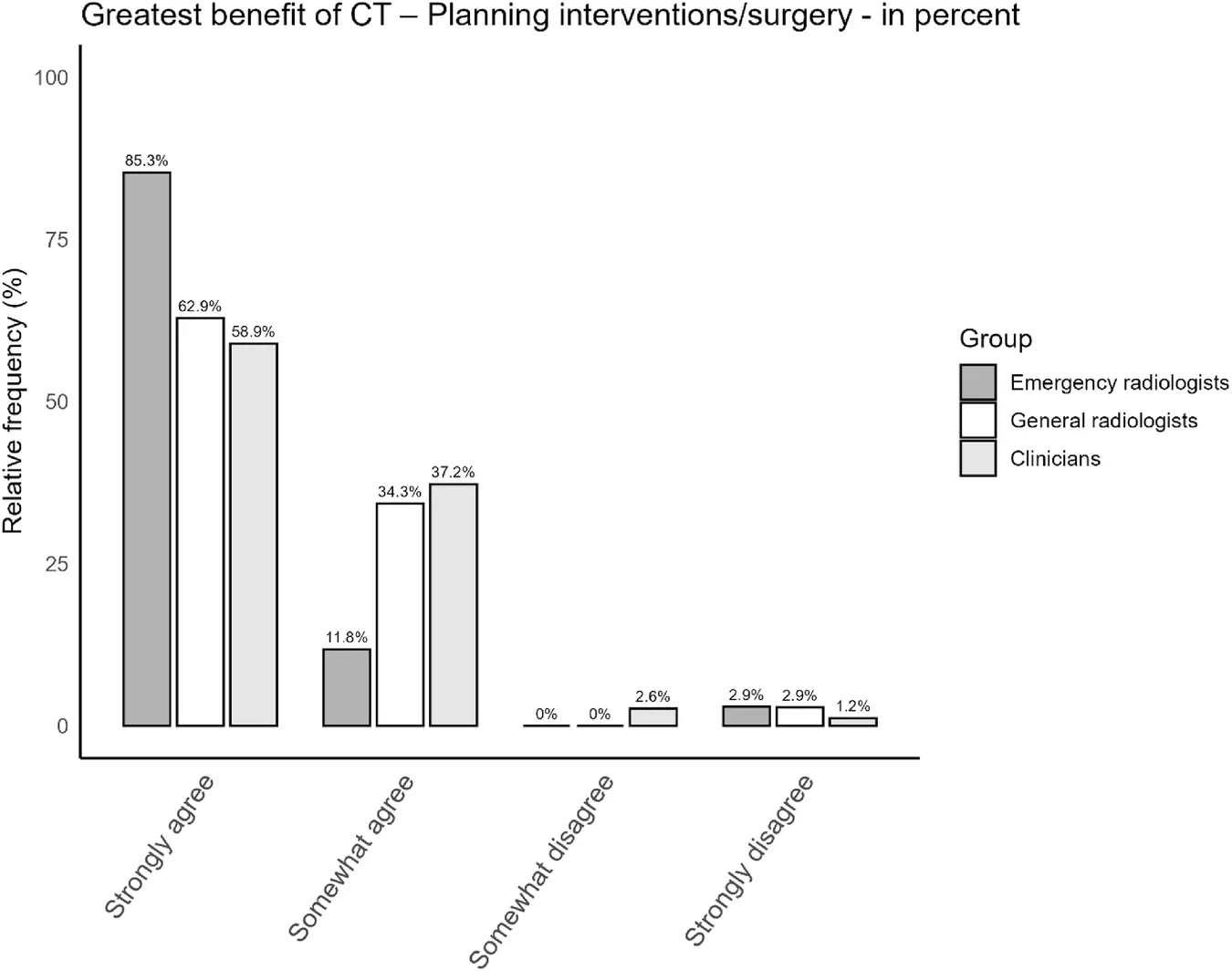
Use of CT for planning radiological interventions or surgery in septic patients

**Fig. 2:**
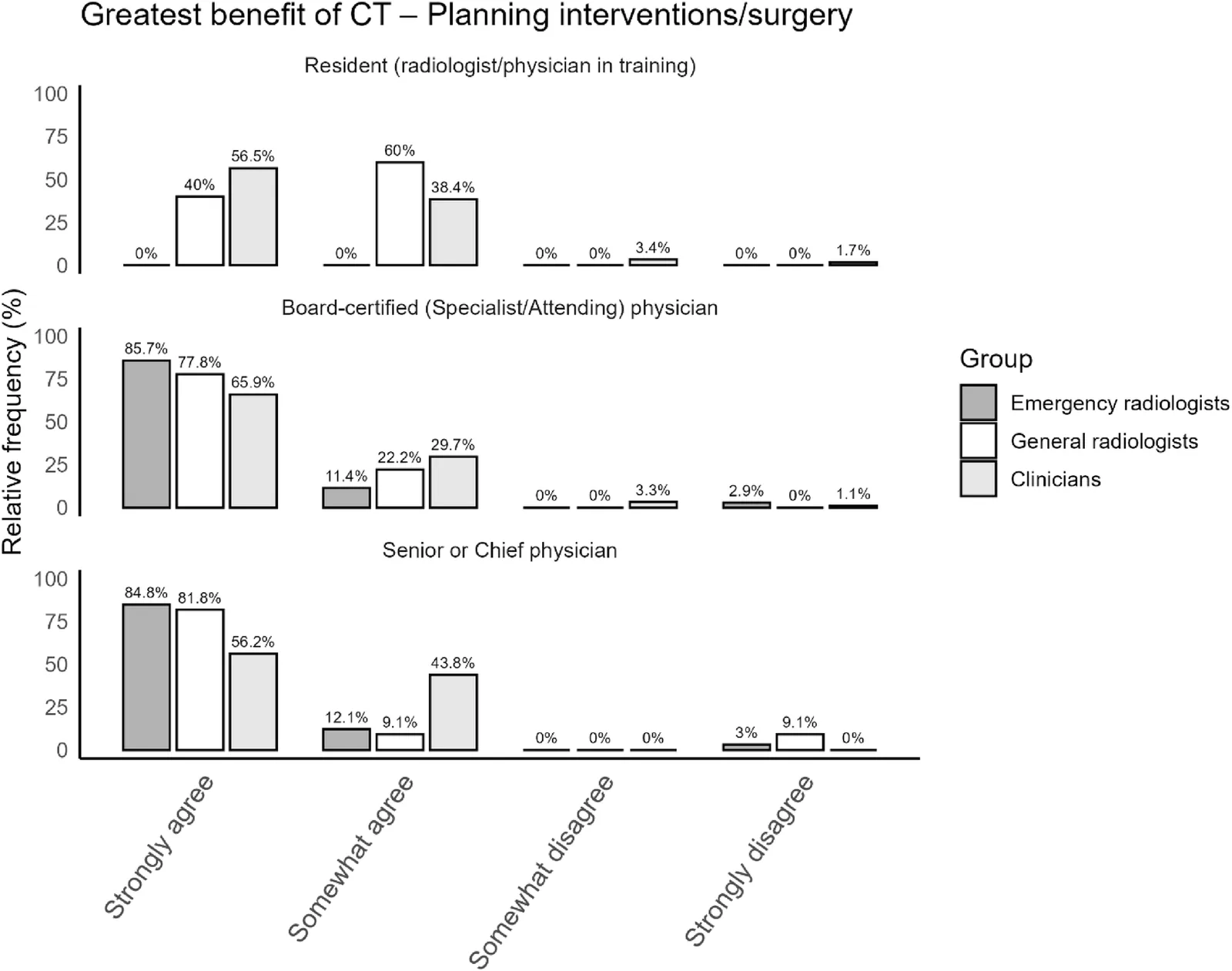
Use of CT for planning radiological interventions or surgery—results of analysis stratified by professional experience based on board-certification and hospital rank

A total of 144 emergency radiologists participated in the ESER survey. Previously collected survey data from clinicians across various specialties and general radiologists were used for comparison, as detailed above, yielding a total of 719 participants for inclusion in the final analysis. 85.3% (*n* = 58) of emergency radiologists participating in the survey strongly agreed that they performed CT in patients with sepsis for planning a radiological intervention or surgery (Fig. 1). There was high agreement between general radiologists (62.9%, *n* = 22, strongly agreed) and clinicians (58.9%, *n* = 201, strongly agreed). Different from emergency radiologists (11.8% (*n* = 8)), a higher proportion of general radiologists somewhat agreed, 32.3% (*n* = 12), and clinicians, 37.2% (*n* = 127). Stratified by professional experience based on board certification and hospital rank revealed the following: the resident group agreed less with the role of CT in planning radiological intervention or surgery as opposed to board-certified physicians or senior/chief physicians (Fig. 2). Of note, there were no resident emergency radiologists (*n* = 0) while the groups of general radiologists and clinicians comprised residents with 60% (*n* = 9) of general radiologist residents opting for “somewhat agree” (while 40% (*n* = 6) of general radiologist residents strongly agreed) and 56.5% (*n* = 100) of clinician residents strongly agreed (while 38.5% (*n* = 68) somewhat agreed). Overall, analysis stratified by professional experience confirmed that most participants strongly agreed that CT played a relevant role in planning interventions or surgery in septic patients.

**Fig. 3:**
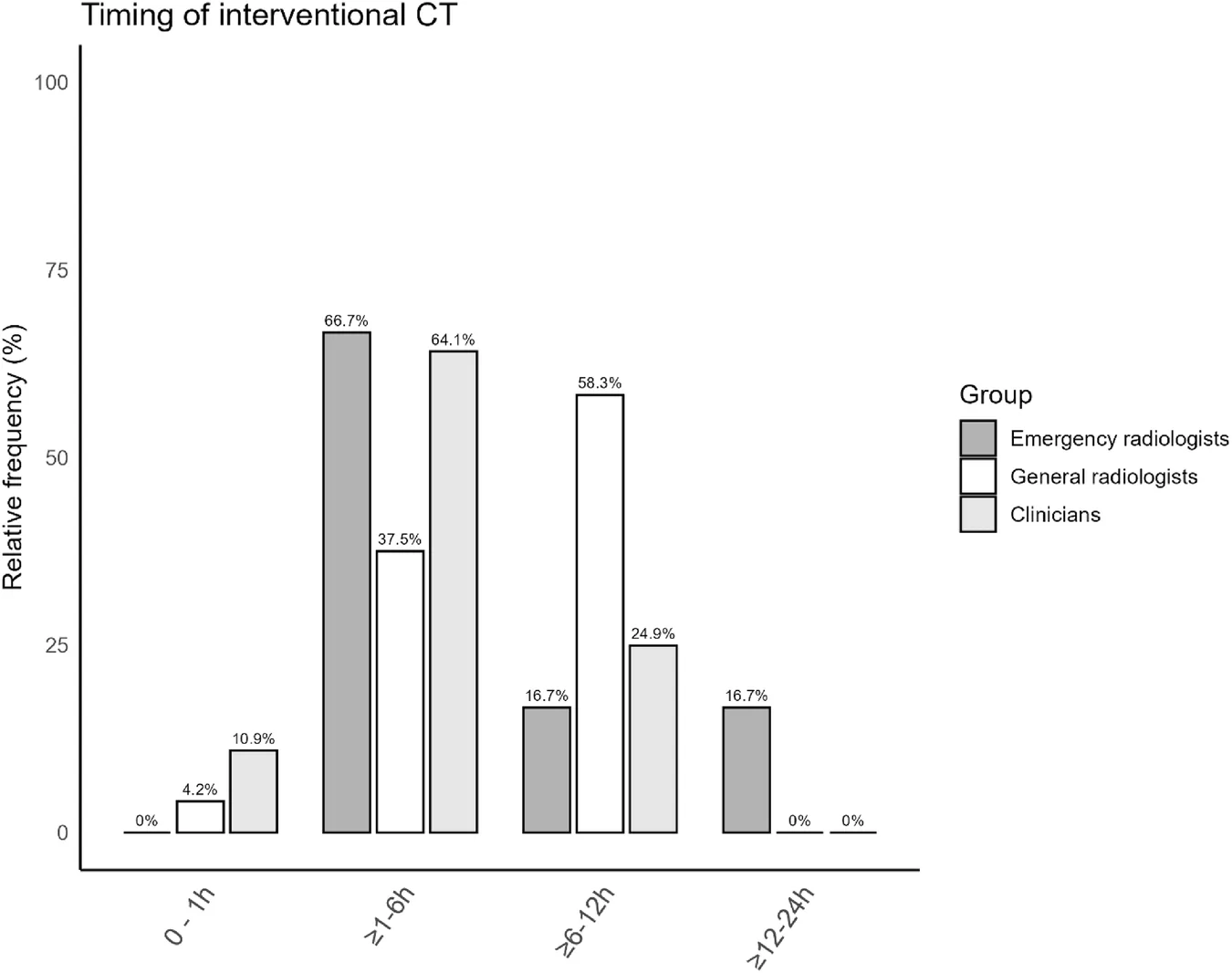
Preferred time window for CT-guided interventions in patients with sepsis

The time window for CT-guided interventions in patients with sepsis, i.e., drainage of a known infectious focus, preferred by most participants, was between 1 and 12 h (Table 1 and Fig. 3). The majority of emergency radiologists (66.7%, *n* = 36) and clinicians (64.1%, *n* = 211) preferred a shorter time window of 1–6 h than general radiologists, who were more inclined to accept a 6–12-h time window (58.3%, *n* = 14).

**Table 1:** Preferred time window of CT-guided interventions for source control in patients with sepsis

Time to CT-guided intervention	Emergency radiologists		General radiologists	Clinicians	Total
0–1 h	0% (*n* = 0)		4.2% (*n* = 1)	10.9% (*n* = 36)	9.1% (*n* = 37)
≥ 1–6 h	66.7% (*n* = 36)		37.5% (*n* = 9)	64.1% (*n* = 211)	62.9% (*n* = 256)
≥ 6–12 h	16.7% (*n* = 9)		58.3% (*n* = 14)	24.9% (*n* = 82)	25.8% (*n* = 105)
≥ 12–24 h	16.7% (*n* = 9)		0% (*n* = 0)	0% (*n* = 0)	2.2% (*n* = 9)
Total	100% (*n* = 54)		100% (*n* = 24)	100% (*n* = 329)	100% (*n* = 407)

χ^2^ = 79.976 · d*f* = 6 · Cramer’s *V* = 0.313 · Fisher’s *p* = 0.000

**Fig. 4:**
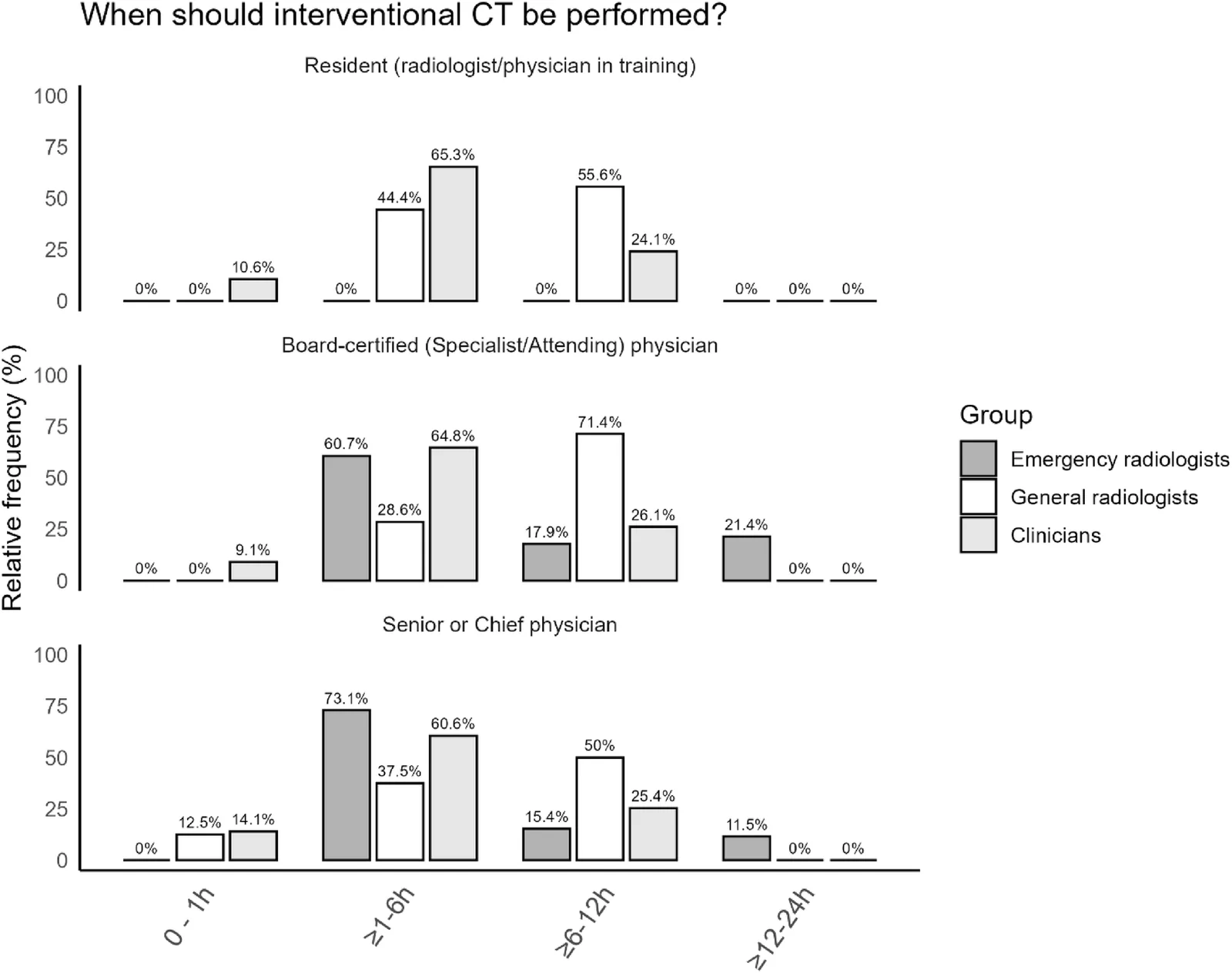
Preferred time window for interventional CT—results of analysis stratified by board certification and hospital rank

Stratification by professional experience revealed no relevant differences in the preferred time window (Fig. 4): there were no residents in the emergency radiologist group (*n* = 0), while the shorter window of 1–6 h was opted for by 60.7% (*n* = 17) of board-certified emergency radiologists and 73.1% (*n* = 19) of senior emergency radiologists. General radiologists tended to opt for a 6–12-h time window (i.e., residents 55.6% (*n* = 5), board-certified 71.4% (*n* = 5), senior 50% (*n* = 4)). Clinicians tended to prefer the 1–6-h window, i.e., residents 65.3% (*n* = 111), board-certified 64.8% (*n* = 57), senior 60.6% (*n* = 43). Controlled for professional experience in years, which showed differences only in the age group below 3 years of experience, while all other age groups behaved similarly in the agreement to the relevance of CT for guiding interventions (Supplementary Fig. 1).

Further differentiation of professional experience related to different age groups revealed minor differences in the distribution of the preferred time window: These were noted among less experienced physicians, i.e., participants with less than 3 years of professional experience (general radiologists, (60% (*n* = 3)) (Supplementary Fig. 2). Emergency radiologists preferred a time window of 1–6 h across all experience levels. Among general radiologists, the higher share of participants who opted for a 6–12-h time window was driven by those with 3–11 years of experience (*n* = 9). Clinicians of all experience levels mainly voted for a 1–6-h time window. Still, one quarter of clinicians with high professional experience of more than 20 years (25.9%, *n* = 7) demanded source control within the first hour.

## Discussion

### Summary

Most respondents in both surveys strongly agreed on the importance of CT for planning interventions in septic patients, particularly emergency radiologists. Preferences for CT-guided intervention timing leaned towards 1–6 h after focus identification among emergency radiologists and clinicians, while general radiologists accepted a later window of 6–12-h. Professional experience did not significantly influence agreement or timing preferences, except for a trend in very experienced physicians who demand very early CT. Overall, our results indicate a strong consensus regarding the role of interventional CT in the management of septic patients, with some minor variations based on specialty and professional experience.

### Literature

To the best of our knowledge, current sepsis guidelines do not offer recommendations on CT-guided interventions for source control [1, 4–7, 13]. As previously mentioned, earlier recommendations suggested the use of imaging to locate infectious sources, while current guidelines lack specific directives regarding the use of CT [1, 4, 5]. Nonetheless, published articles suggest that CT plays a pivotal role in verifying suspected infection sites and uncovering alternative foci that might necessitate surgical or interventional management [8–14].

Scientific evidence regarding CT-guided interventions for managing infectious foci in patients with sepsis is still limited. We previously assessed perspectives of physicians from various specialties regarding the use of CT in patients with sepsis [17, 19]. These data support the notion that physicians from various backgrounds consider timing of CT a relevant aspect of patient management. In the present study, we reported perspectives on CT-guided interventions of emergency radiologists and compared them with those of general radiologists and clinicians from a previous survey. It is relevant to understand different specialties’ perspectives and requirements in interdisciplinary communication for improving the service that emergency radiology provides in patients with sepsis. Interestingly, emergency radiologists and clinicians were aligned on the relevance of a shorter time window (1–6 h) for CT-guided interventions as compared with general radiologists. Professional experience alone was insufficient to explain these differences in timing, though a trend for immediate CT-guided intervention was seen in the small subgroup of physicians of > 20 years (general radiologists and clinicians). Still, the observed difference in timing of interventional CT between emergency radiologists and general radiologists—with emergency radiologists leaning towards a shorter time window—requires further investigation. We hypothesize that the more common exposure to sepsis cases and regular discussions with involved clinicians may alert emergency radiologists that patients with sepsis potentially benefit from rather prompt source control. Importantly, the heterogeneity of participating countries may account for regional variations in emergency radiology practices. These data will inform the planning of consensus meetings to improve interdisciplinary alignment on CT-guided interventions in sepsis.

### Limitations

Data obtained from the multi-center survey of emergency radiologists (ESER) were compared with data from a single-center survey using a previously established questionnaire, i.e., the comparisons are fundamentally limited by the design of this study [17, 19]. While emergency radiologists’ perspectives are central in this manuscript, we used data from the earlier single-center survey as a reference. Specifically, these were categorized into two distinct groups: general radiologists and clinicians. Even though the results presented in this manuscript stem from two sequential surveys, it is important to note that only minor adjustments were made to the questionnaire. Also, general radiologists from the tertiary care facility might have some experience in emergency imaging, e.g., from rotations during their residency training. Still, general radiologists could be seen as less proficient than dedicated emergency radiologists. As no ER specialty has been established as of now, we used ESER membership to identify emergency radiologists for the survey presented here. Importantly, the data for emergency radiologists were collected through a multinational survey involving various centers, whereas the results for general radiologists and clinicians of various specialties were obtained at a single center. Importantly, the clinicians that serve as a comparison here are a heterogeneous group, as shown previously—a more detailed analysis was not intended in this manuscript. Additionally, there are differences in professional experience among the physician groups surveyed, with ESER radiologists typically having more experience, which may have affected our results. This factor has been accounted for in this study by conducting a stratified analysis of professional experience based on both board certification and years of professional experience. Lastly, not every question was answered by all participants, which may reduce the validity of some items. The statistical approach has been chosen accordingly, so to reduce bias in analyzing all responses and not lose relevant participant feedback due to the exclusion of questionnaires that lack one or more answers. Given the small sample size and the exploratory nature of this study, the authors opted for a primarily descriptive statistical approach to avoid potentially misleading inferences from formal hypothesis testing. Also, some of the subgroups analyzed in detail comprised very low numbers, therefore limiting generalizability. The role of interventions such as both abscess drainage in non-septic patients and subgroups in patients with sepsis and septic shock is relevant but beyond the scope of this manuscript. A more detailed analysis of different specialties in the clinicians’ subgroup was beyond the scope of this manuscript and has been published elsewhere [17]. These data provide limited evidence for treatment preferences in sepsis that require replication.

## Conclusions

The ESER survey presented here reveals a robust consensus among radiologists regarding the critical role of CT in planning interventions in septic patients, with emergency radiologists showing the strongest agreement. Additionally, while the preferred timing of interventions for source control varies slightly among specialties, significant emphasis is placed on prompt CT-guided interventions, ideally within the first six hours after focus detection. The data from this hypothesis-generating survey suggest a consensus on the timing of CT-guided interventions that requires confirmation from another prospective study.

## Supplementary information

ELECTRONIC SUPPLEMENTARY MATERIAL

## Data Availability

Data will be shared by the corresponding author upon reasonable request.
