# Supplementary material for: Planning and performing CT-guided interventions for source control in septic patients: a survey by the European Society of Emergency Radiology
Source: Insights Imaging. 2026 Sep 15;17:228. doi: 10.1186/s13244-026-02374-6 (PMC13578189; doi:10.1186/s13244-026-02374-6)
Supplement: Supplementary file 1 [file 13244_2026_2374_MOESM1_ESM.pdf]

Planning and performing CT-guided interventions for source control in septic patients: A survey by the European Society of Emergency Radiology

ELECTRONIC SUPPLEMENTARY MATERIAL

Supplemental Figures

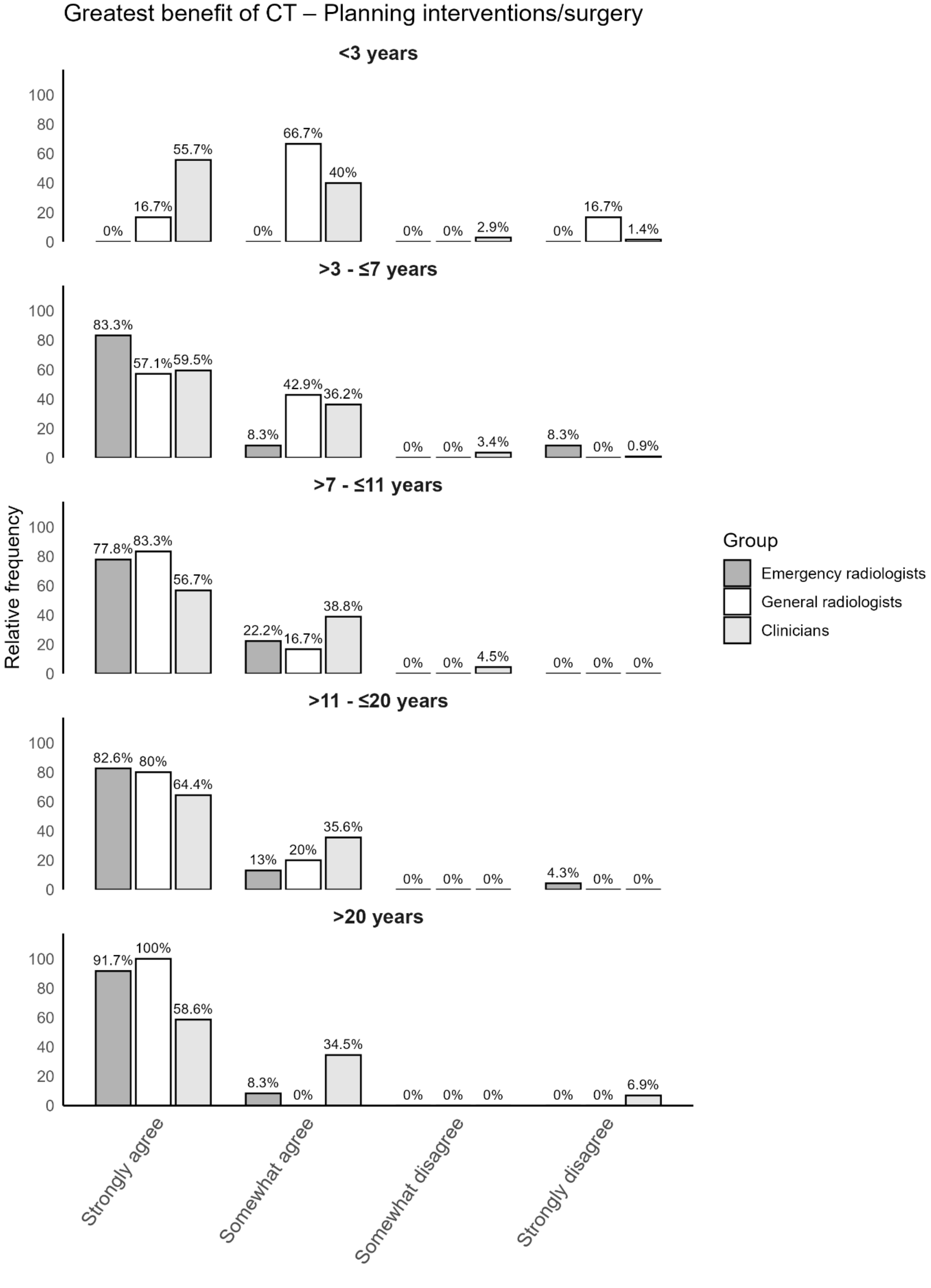

Suppl. Figure 1: Use of CT for planning radiological interventions or surgery – results of subgroup analysis based on years of professional experience.

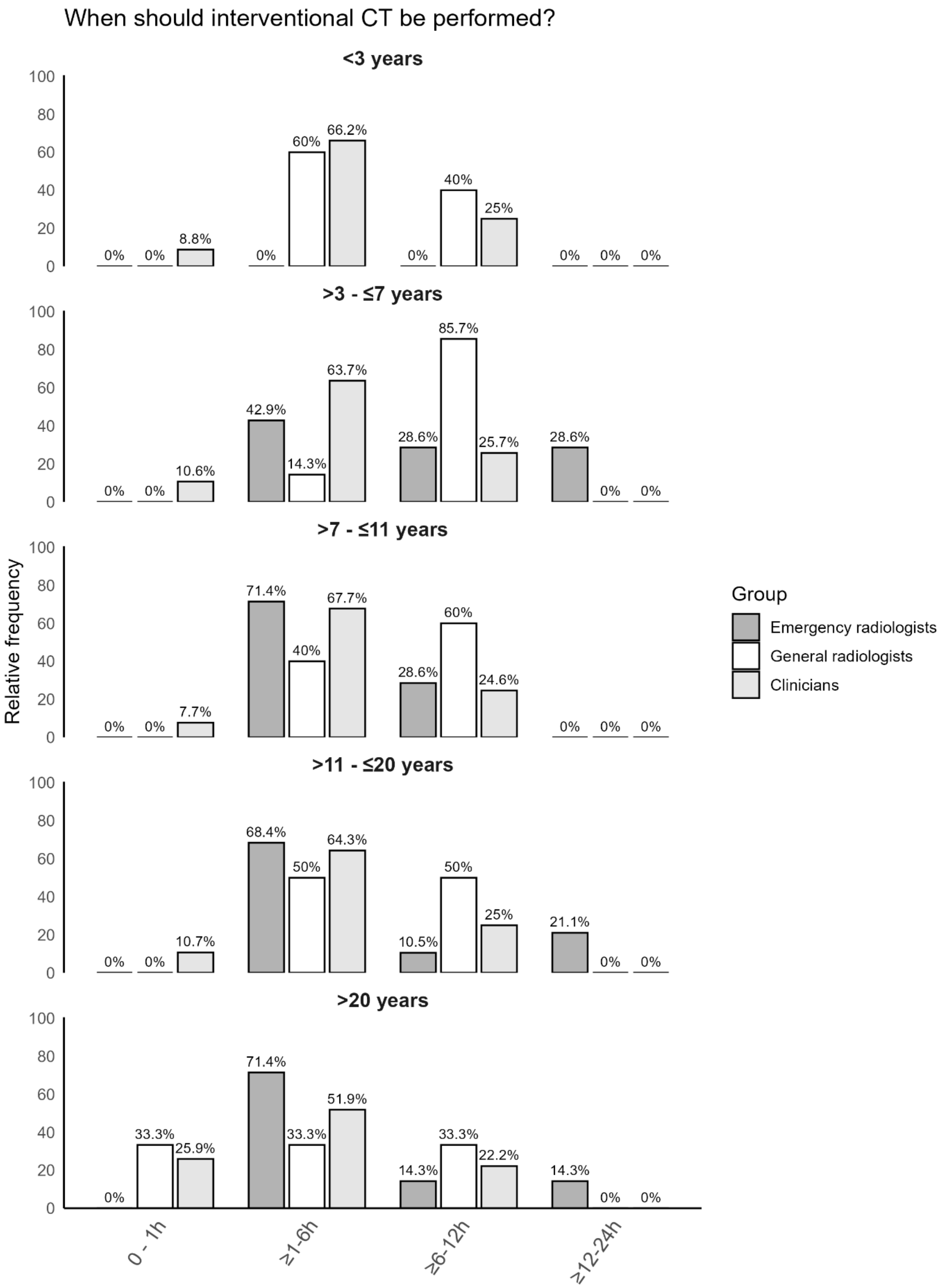

Suppl. Figure 2: Timing of CT for planning radiological interventions or surgery – results of analysis stratified by professional experience in terms of years of experience.
